# A Modern Multidisciplinary Method to Characterize Natural White Spot Lesions with 2D and 3D Assessments: A Preliminary Study

**DOI:** 10.3390/jpm14050542

**Published:** 2024-05-19

**Authors:** Flavia Vitiello, Giulia Orilisi, Valentina Notarstefano, Michele Furlani, Nicole Riberti, Tiziano Bellezze, Florence Carrouel, Angelo Putignano, Giovanna Orsini

**Affiliations:** 1 Department of Clinical Sciences and Stomatology (DISCO), Università Politecnica delle Marche, 60126 Ancona, Italy; f.vitiello@pm.univpm.it (F.V.); g.orilisi@pm.univpm.it (G.O.); m.furlani@pm.univpm.it (M.F.); a.putignano@univpm.it (A.P.); 2 Health, Systemic, Process (P2S), Research Unit UR 4129, University Claude Bernard Lyon 1, 69008 Lyon, France; florence.carrouel@univ-lyon1.fr; 3 Department of Life and Environmental Sciences, Polytechnic University of Marche (DISVA), Via Brecce Bianche, 60131 Ancona, Italy; v.notarstefano@univpm.it; 4 Department of Neurosciences Imaging and Clinical Sciences (DNISC), University of Chieti-Pescara, 66100 Chieti, Italy; nicole.riberti@unich.it; 5 Department of Materials, Environmental Sciences and Urban Planning (SIMAU), Università Politecnica delle Marche, 60131 Ancona, Italy; t.bellezze@univpm.it; 6 National Institute of Health and Science of Aging (INRCA), 60124 Ancona, Italy

**Keywords:** white spot lesion, enamel, microhardness, Raman microspectroscopy, microcomputed tomography, SEM-EDX

## Abstract

In this preliminary study, a multidisciplinary method based on high-resolution analytical techniques (such as microcomputed tomography, Raman Microspectroscopy, scanning electron microscopy, and Vickers microhardness test) was exploited to evaluate the alterations that occur in human teeth at the initial stage of the carious lesion. To this purpose, six extracted molars displaying a natural white spot lesion (WSL) were investigated. Specific morphological, structural, and chemical parameters, such as the mineral density, indentation hardness, molecular and elemental composition, and surface micromorphology were obtained on the WSL, and the results were statistically compared (*t*-test, *p* < 0.05) to those of the sound enamel on the same tooth. In the WSL, with respect to the sound area, a decrease in the mineral density and crystallinity was detected together with differences in the molecular composition and surface microstructure, such as the occurrence of micropores and irregularities. Moreover, the elemental analysis highlighted in WSL showed a statistically significant decrease in Ca and P percentages. In conclusion, this multidisciplinary approach allows us to fully characterize the area of interest, providing a deeper knowledge of these enamel lesions, which could have important clinical implications.

## 1. Introduction

Over time, teeth can be subjected to enamel demineralization processes, and this decrease in mineral content could lead to greater vulnerability to tooth decay and dental damage. This phenomenon can derive from genetic, systemic, or environmental causes, leading to the formation of developmental defects [1]. The first clinical phase of the carious lesions is characterized by the initial demineralization of the enamel subsurface without cavitation, leaving an apparently intact or pseudo-intact surface layer that covers the mineral-free area. Cavities are formed as a result of the partial dissolution of carbonated hydroxyapatite (HA) crystallites due to cariogenic biofilm bacteria that metabolize dietary carbohydrates and produce organic acids, resulting in an imbalance between demineralization and remineralization cycles [2,3]. As a consequence of the significant difference in the refractive index of the medium within the acid-created pores in the demineralized area, a whitish opaque lesion becomes visible, called a white spot lesion (WSL) [4]. This chalky appearance represents an optical phenomenon caused by mineral loss in the subsurface and the surface of the enamel [5]: in fact, the surface layer in natural WSLs has a mean thickness of 40–45 μm and a mineral content of 82–84% [3].

It is known that the crystal structure of HA is subject to a natural cycle that involves demineralization and remineralization processes. This cycle works in favor of either demineralization or remineralization, depending on environmental factors [6]. Hence, the progression of the carious lesion can be prevented by shifting the reactions toward remineralization. However, as demineralization continues, there is a loss of integrity on the enamel surface, which can become cavitated [7,8]. In 1966, Dirks was the first to describe the possible remineralization of natural enamel lesions formed in vivo as a normal occurrence in the oral environment [9]. Indeed, saliva can remineralize these lesions to some degree, but this is a slow process that rarely results in a complete resolution of the lesions [10]. Otherwise, if the tooth lesion is untreated, the acid environment continues to spread into the porous subsurface enamel, dissociating and producing hydrogen ions [11]. These acid ions, in turn, trigger the release of calcium and phosphate out of the tooth in the oral cavity. 

The main goal of modern dentistry is to develop early-stage and minimally invasive treatments for dental lesions. In light of this, the development of a reliable analytical approach able to provide high-resolution information on the pathological structure of the enamel is mandatory to thoroughly study initial carious lesions and evaluate the effectiveness of remineralizing agents to replenish the lost crystalline structure. 

In the dental research field, various analytical techniques can be exploited, each able to provide detailed information on a particular aspect (mechanical, morphological, molecular, and so on). Raman Microspectroscopy (RMS) represents a valuable and innovative tool to in vitro evaluate hard dental tissues. In fact, this vibrational technique has the advantage of simultaneously providing a morpho-chemical correlation between the microscopic information obtained through the visual analysis of the sample and its chemical and macromolecular structure [12,13]. On the other hand, microcomputed tomography (µ-CT) is a powerful, non-destructive instrument for examining the demineralization and remineralization processes of teeth, which is valuable for testing the effectiveness of carious lesion treatments [14]. It provides qualitative and quantitative information on the microstructure of materials with a micron-level spatial resolution while also allowing the evaluation of defects and differences in density and morphology [15]. Currently, Scanning Electron Microscopy (SEM) represents a common method in dental research to investigate not only the microstructure and morphology of sound and pathological teeth but also the structural features of dental materials [16,17]. During SEM observations, the elemental composition of a given material can also be obtained [18] by means of Energy-Dispersive X-ray Spectroscopy (EDX). The identification and the topographical distribution of different chemical elements can provide reliable information on the specimen surface [19]. Finally, the Vickers microhardness test (VMH) is a useful tool to measure the mechanical properties of hard dental tissues [20].

Based on this evidence, a preliminary study based on a multidisciplinary high-resolution approach, coupling RMS, µ-CT, VMH, and SEM-EDX analyses, has been exploited for characterizing the chemical and microstructural features of WSLs in comparison to sound enamel. The encouraging results obtained from this 2D and 3D investigation could pave the way for the development of a new analytical method that is useful for investigating pathologies that affect teeth. 

## 2. Materials and Methods

### 2.1. Sample Collection

Permanent human molars (*n* = 6) were collected at the Department of Clinical Sciences and Stomatology of the Università Politecnica delle Marche (Ancona, Italy). Teeth were surgically extracted for therapeutic purposes, and informed consent was signed by patients, ensuring they were fully aware that their hard dental tissues would be used for research purposes, according to the Local Ethical Committee guidelines and the WMA–Declaration of Helsinki (2018) (WMA–The World Medical Association Declaration of Helsinki–Ethical Principles for Medical Research Involving Human Subjects). After surgical extraction, samples were immersed in an ultrasonic bath with distilled water for 3 min in order to remove blood and biological remains [21,22]. All tooth samples were carefully examined by two experienced clinicians (F.V., G.Ori.) according to the following inclusion and exclusion criteria. The inclusion criteria were as follows: (i) teeth were permanent human molars, and (ii) the WSL was in the proximal–vestibular zone. The exclusion criteria were as follows: (i) the presence of cavitated caries lesions, including hypoplastic defects and enamel cracks, and (ii) the presence of previous treatment.

According to the criteria of the principal carious lesion classification [23], WSLs were defined as primary active incipient enamel caries lesions located on the smooth surface of adult patients.

Samples were stored in artificial saliva (Biotene Oralbalance Gel, GSK, London, UK), which was changed daily until measurements were undertaken by RMS and µ-CT analyses. Then, teeth were embedded in epoxy resin blocks (Technovit, Kulzer Technik, Wehrheim, Germany), leaving the WSL area exposed for VMH and SEM-EDX analyses [24]. 

### 2.2. RMS Measurements and Data Analysis

The XploRA Nano Raman Microspectrometer (Horiba Jobin-Yvon GMBH, Oberursel, Germany), equipped with a 785-nm diode laser, was used as a source. All RMS measurements were acquired using a 5× objective (Olympus, Tokyo, Japan). The spectrometer was calibrated to the 520.7 cm^−1^ line of silicon prior to spectral acquisition. A 600 lines per mm grating was chosen. A 200 μm confocal pinhole was used for all measurements. The spectra were dispersed onto a 16-bit dynamic range Peltier-cooled CCD detector. On each tooth sample, a Raman map was acquired on a rectangular area (241.5 µm × 167 µm) at the interface between the WSL and the sound enamel. A step size of ~15 µm was adopted for a total number of 192 spectra. 

On each Raman map, the following values were calculated: the area (A) of the band centered at 1660 cm*^−^*^1^ (spectral range 1655–1707 cm*^−^*^1^), representing the Amide I band of proteins (A_1660_) [25,26]; the area (A), intensity (I), and full width at half maximum (FWHM) of the band centered at 960 cm*^−^*^1^ (spectral range 929–976 cm*^−^*^1^), assigned to the stretching of PO_4_^3−^ groups in HA (A_960_, I_960_, and FWHM_960_) [27,28]; and the intensity (I) of the band centered at 1070 cm*^−^*^1^ (spectral range 1051–1088 cm*^−^*^1^), assigned to the stretching of CO_3_^2−^ groups (I_1070_) (Labspec 6 software, Horiba Scientific, Irvine, CA, USA) [27,29,30]. These parameters were used to generate false-color images showing the spatial distribution of the following spectral features: I_960_, A_960_/A_1660_ (Mineral/Matrix), FWHM_960_ (inverse of Crystallinity), and I_1070_/I_960_ (carbonate/phosphates) [29,31]. 

Finally, from each Raman map, spectra were extracted and submitted to preprocessing procedures, including baseline correction (2 iterations of the polynomial method), smoothing (5 points), and vector normalization (OPUS 7.5 software).

### 2.3. µ-CT Acquisition and Reconstruction

Each individual sample underwent scanning using a µ-CT system Bruker SkyScan 1174 (SkyScan-Bruker, Antwerp, Belgium), installed at the CISMiN Laboratories of the Università Politecnica delle Marche (Ancona, Italy). The projection settings used were as follows: an acceleration voltage of 50 kV; a beam current of 800 μA; an aluminum filter with a thickness of 1 mm; a pixel size of 11.5 μm, and a rotation of 180° in 0.3° step with an exposure time of 10 s per projection. On average, the scanning process took approximately 2 h. A total number of 830 reconstructed sections were obtained for each sample, providing axial information covering an approximate tooth thickness of 12 mm. To convert tooth projections into cross-sectional slices, the NRecon software (Version 1.6.10.2, Bruker, Billerica, MA, USA) was used, employing the following correction settings: Ring artifacts (7.0); smoothing (6.0); beam hardening (40%); and proper misalignment compensation. 

**Figure 1:**
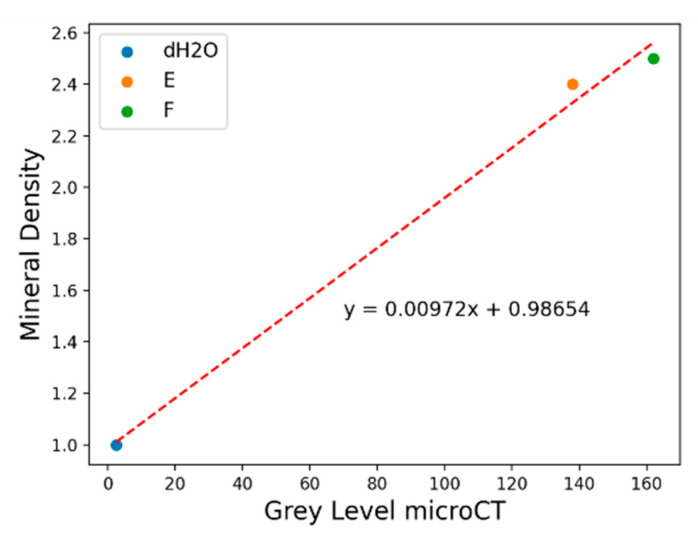
Fitting curve used to determine MD starting from gray values acquired from phantom images: H_2_O (distilled water = 1 g/cm^3^), composite E (2.4 g/cm^3^), and composite F (2.5 g/cm^3^).

The mineral density (MD) was quantified following the protocol proposed by Schwass et al. [32]. The MD was calculated by the equation MD = 0.00972x + 0.98654 (Figure 1). In order to preserve the grey scale levels and the related MD values, the original cross-section sagittal image was de-noised. The curve (Figure 1) used to convert gray values read in the acquisition into MD values (expressed in g/cm^3^) was obtained by fitting gray values from three phantoms used for the calibration in the same setting configuration. The phantoms used were as follows: distilled water (1 g/cm^3^), Herculite XRV Ultra (Kerr, Bolzano, Italy) (2.4 g/cm^3^), and Premise Flowable (Kerr, Bolzano, Italy) (2.5 g/cm^3^). Grey values, ranging from 0 to 255, were evaluated using the histogram function in square regions inside each portion (enamel, dentin, and WSL); the analysis was performed on four different slices using Fiji [33]. ORS Dragonfly software Version 2022.1 for Windows (Object Research Systems (ORS) Inc., Montreal, QC, Canada) was used to visualize the 3D reconstruction image and to analyze the different tooth portions (WSL, Enamel, Dentin) [34].

### 2.4. Microhardness Evaluation

The VMH test was conducted by means of a Remet microhardness tester HX-1000TM (Remet S.A.S., Casalecchio di Reno, Italy). For each sample, the specific areas of both the WSL and the sound enamel were chosen. Three indentations were made on each selected area using a pyramid-shaped diamond indenter, with a load of 100 g applied for 15 s. The Proximo ver. 9 software was employed to measure the diagonals of the imprints, which were used to calculate the Vickers microhardness number (HV number), expressed as Kg/mm^2^. The surface microhardness was measured at three points in the testing areas, and the mean value was calculated and reported.

### 2.5. SEM-EDX Evaluation

SEM observations were performed using a Zeiss Supra 40 field-emission electron microscope (Zeiss, Oberkochen, Germany) (Centre for Electron Microscopy (CISMIN) Department of SIMAU, Università Politecnica delle Marche, Ancona, Italy). All samples were carefully cleaned, dehydrated, and then placed onto a sample holder before being exposed to metallization by the vacuum precipitation of a thin gold film on the dental surface. SEM micrographs of the enamel were captured at magnifications of 2000× and 8000× using an SEM operating at 20 kV and a 7 mm working distance. The resulting micrographs were used to assess the micromorphology and elemental composition of both the sound enamel and WSL [35]. The surface elemental characterization was conducted using EDX with EDAX Element Microanalysis (AMETEK Gmbh, EDAX Business Unit, Weiterstadt, Germany) [36]. EDX analysis was performed on three distinct areas of the samples using the following operating parameters: 15 mm working distance, 20 kV acceleration voltage, and 1000× magnification. The degree of mineralization was evaluated by quantifying the amount of phosphorus (P) and calcium (Ca) present and calculating the ratio Ca/P [37]. 

### 2.6. Statistical Analysis

Statistical analysis was performed using the software package Prism 6.0 (GraphPadSoftware, San Diego, CA, USA). All data were presented as the mean ± standard deviation. Statistical significance among groups was evaluated using Student’s *t*-test. Statistical significance was set at *p* < 0.05 (*, *p* < 0.05; **, *p* < 0.01; ***, *p* < 0.001, and ****, *p* < 0.0001).

## 3. Results

### 3.1. RMS Results

**Figure 2:**
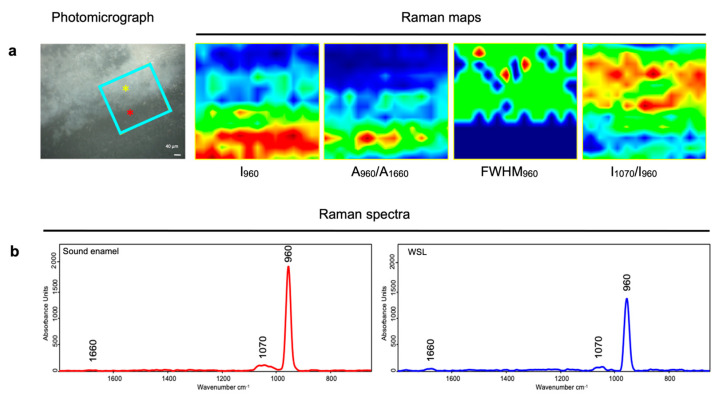
(**a**) Raman mapping analysis of a representative area at the interface between WLS and sound enamel. Photomicrograph with the mapped area (light blue box, 241.5 µm × 167 µm; yellow and red asterisks indicate the areas with WSL and sound enamel, respectively) and false color images show the topographical distribution of the following spectral parameters: I_960_ (phosphates), A_960_/A_1660_ (Mineral/Matrix), FWHM_960_ (inversely proportional to Crystallinity), and I_1070_/I_960_ (carbonates/phosphates). Different color scales were used for the better interpretation of the data: black/blue color corresponds to the lowest values, and green intermediate and red/dark red to the highest ones. (**b**) Raman spectra collected on sound enamel (red line) and WLS (blue line) (spectral range 1800–650 cm^−1^).

**Figure 3:**
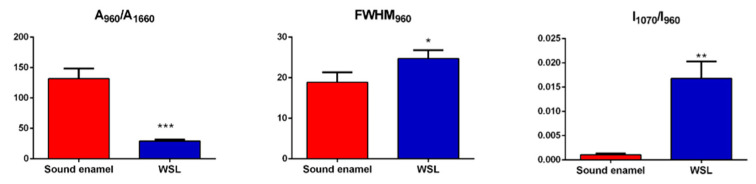
Statistical analysis of the following spectral parameters: A_960_/A_1660_ (mineral/matrix), FWHM_960_ (inversely proportional to crystallinity), and I_1070_/I_960_ (carbonates/phosphates). Data are presented as the mean ± standard deviation. Statistical significance among groups was evaluated using Student’s *t*-test. Statistical significance was set at *p* < 0.05 (*, *p* < 0.05; **, *p* < 0.01; ***, *p* < 0.001).

In Figure 2a, the photomicrograph of a representative tooth sample is shown with the light blue box representing the area at the interface between the WSL (yellow asterisk) and the sound enamel (red asterisk) on which the Raman map was collected. Marked differences in the chemical composition between the lesion and the sound enamel are displayed by the false color images, which report the topographical distribution of specific spectral parameters. In detail, in WLS, a lower number of phosphate groups (as shown by the I_960_ and A_960_/A_1660_ maps) was observed, together with a minor crystallinity value (inverse of the FWHM_960_ map) and a higher amount of carbonates with respect to phosphates (I_1070_/I_960_ map). These findings were also confirmed by the analysis of the Raman spectral profiles (Figure 2b), in which the band at 960 cm^−1^, representative of PO_4_^3−^ groups in HA, was lower in height in WSL with respect to sound enamel and by the statistical analysis of the above-mentioned parameters (Figure 3). 

### 3.2. µ-CT Results

**Figure 4:**
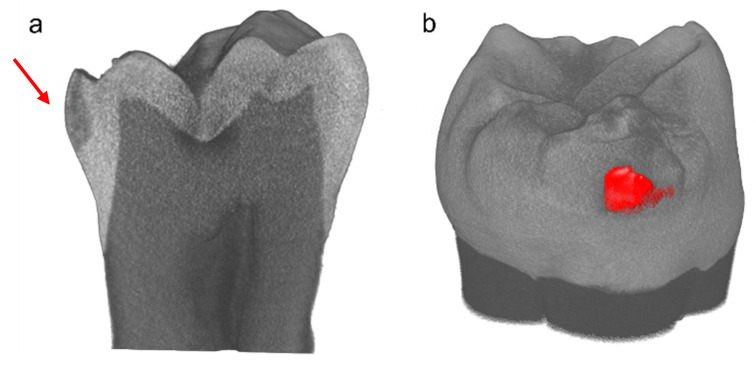
(**a**) Sagittal cross-section image of reconstructed µ-CT image of the representative sample. WLS appeared as a slightly darker triangular-shaped region on the left side of the image (red arrow). (**b**) The red color was used to better highlight the differences in MD.

As regards µ-CT analysis, a sagittal slice of a representative tooth sample with WSL is shown in Figure 4a. The lesion, located in the external margin of the enamel (as indicated by the red arrow) with a lunar shape, displayed a different MD with respect to the sound enamel (as highlighted by the color image in Figure 4b). The MD obtained from the µ-CT analysis and the corresponding grey scale values of enamel, dentin, and WSL are reported in Table 1. The MD of the sound enamel was 2.52 g/cm^3^ ± 0.005, in accordance with the scientific literature, while dentin showed an MD value of 1.91 ± 0.008 g/cm^3^. As regards WSL, the extent of the MD reduction varied not only between lesions but also across different points within the same lesion [38]; however, the lowest value was 2.12 g/cm^3^, while the mean value was 2.14 ± 0.021 g/cm^3^. 

### 3.3. VMH Results

**Figure 5:**
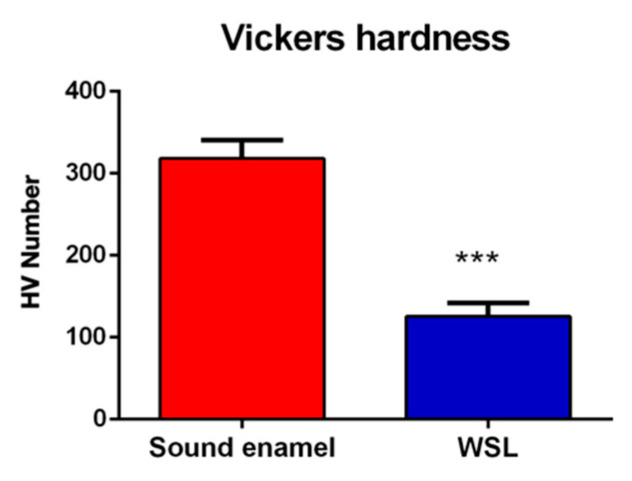
Microhardness values, expressed as the mean HV number (Kg/mm^2^) ± standard deviation, of sound enamel and enamel with WSL. Statistical significance between groups was evaluated using Student’s *t*-test. Statistical significance was set at *p* < 0.05 (***, *p* < 0.001).

The VMH test carried out on WSLs and sound enamel revealed a statistically significant difference (*p* < 0.0001) between the surfaces analyzed (Figure 5). Hardness was in the range of 351–333 kg/mm^2^ for the sound enamel and 117–124 kg/mm^2^ for the enamel with WSL.

### 3.4. SEM-EDX Results

**Figure 6:**
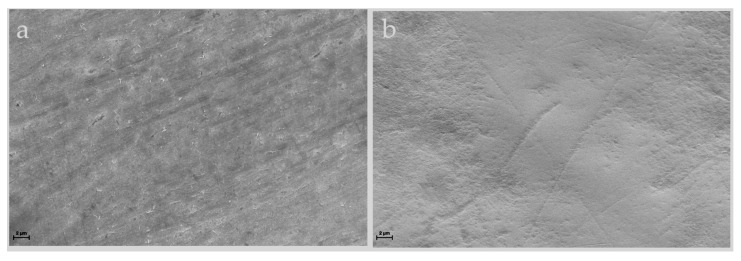
Scanning electron micrographs of sound enamel collected at 2000× (**a**) and 8000× (**b**) magnification, respectively. SEM images show the surface micromorphology of sound enamel with the typical intact regular structure.

**Figure 7:**
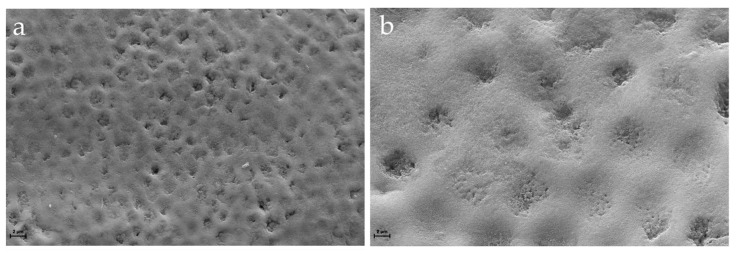
Scanning electron micrographs of a representative WSL collected at 2000× (**a**) and 8000× (**b**) magnification, respectively. SEM images highlight the partial dissolution of the apatite crystals showing micropores and irregularities of the demineralized enamel.

SEM images displayed different surface micromorphologies of the enamel. Figure 6 shows the intact smooth surface with the regular pattern of the sound enamel and preserved crystalline structure anatomy, while Figure 7 presents an irregular enamel surface of the WSL with typical honeycomb morphology showing the partial dissolution of prismatic and interprismatic areas.

**Figure 8:**
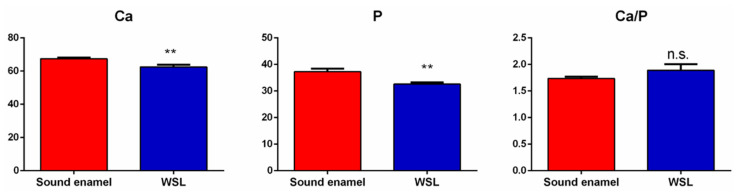
EDX results of Ca (at %), P (at %), and Ca/P ratio of sound enamel and enamel with the WSL. Statistical significance among groups was evaluated using Student’s *t*-test. Statistical significance was set at *p* < 0.05 (**, *p* < 0.01; n.s.: non-statistically significant).

The elemental analysis of the samples is shown in Figure 8. EDX highlighted that Ca and P (percentage, %) are the main elements both in sound enamel and WSL. Noteworthy, in the area of the lesion, a statistically significant reduced amount of Ca and P was observed, albeit maintaining a similar and non-statistically significant Ca/P ratio.

## 4. Discussion

WSLs in enamel are a topic of considerable interest as they represent the initial stage of the development of a carious lesion. Despite the prevalence of these lesions, their etiology, structure, and mineral gradient densities are still poorly elucidated.

In the present study, WSLs were analyzed by exploiting different high-resolution analytical techniques, including RMS, µ-CT, VMH, and SEM-EDX. High-quality 2D and 3D data regarding the chemical, morphological, and mechanical features of these early-stage enamel lesions have been reported [38].

Understanding the distribution of minerals in calcified tissues is highly significant. Indeed, the MD has been widely recognized as a fundamental parameter for assessing the degree of demineralization and remineralization in dental caries. It offers valuable insights into the dynamic changes occurring in the 3D spatial distribution pattern of minerals within carious lesions [39]. Although the µ-CT has been applied to the study of teeth since 1989 [40], little progress has been made in terms of calibration standards over the ensuing 30 years. The scarcity of information currently available on mineral concentration and distribution reflects the complexity of this issue. 

In this study, to standardize the MD calculation, the equation shown in Figure 1 was obtained using three different phantoms with known density [32,41]. The calculated MD of sound enamel was 2.52 g/cm^3^, which is within the range of 2.37–3.10 g/cm^3^ reported in the literature [38,42]. Conversely, the MD of WSLs was ca. 2.14 g/cm^3^, which is, thus, significantly lower with respect to the value of the sound enamel and below the lower end of the mentioned range. These results are consistent with the findings of other authors [38,42]. Since some variability in the MD could be observed in different areas of the same teeth, both in sound and hypomineralized enamel, RMS was exploited to confirm such results. The identification of specific RMS markers representative of sound and pathological hard dental tissues can allow the diagnosis of several dental diseases to improve and detect early-stage dental lesions [13].

The Raman bands observed in the sound enamel are in line with those reported in the scientific literature [12,43]. The lower MD values of WSLs, compared to sound enamel, were associated with a reduction in the bands at 960 cm^−1^ and 1070 cm^−1^, related to phosphate and carbonate groups, respectively, and with an increase in the organic matrix, corresponding to a decrease in the mineral/matrix ratio (A_960_/A_1660_ ratio). These findings were also confirmed by the RMS mapping of the WLS–sound enamel interface. In particular, regarding the carbonate/phosphate ratio (I_1070_/I_960_ ratio), higher values were found in WSLs due to a higher quantity of carbonate groups and lower presence of phosphate with respect to sound enamel. These findings allow us to hypothesize the occurrence of some alterations in the size and shape of enamel crystals, leading to the compromised integrity of the apatite structure within the enamel prism. In fact, the false-color image generated from the ratio of carbonates to phosphates, which is used to analyze changes in the inorganic components of enamel in each zone of the lesion, showed an elevated ratio in the lesion area compared to sound enamel. 

During the carious process, the enamel prisms are submitted to a partial demineralization, generally at the cores, creating gaps in the interrod region [44]. This caused a decrease in the crystallinity of WSL with respect to sound enamel. In this study, only a slight decrease in crystallinity was found in WLSs. Considering that this parameter could be related to the degree of progression in the carious process [45,46], the slight decrease observed in the interface of WLS/sound enamel can allow us to hypothesize that the analyzed lesions represent the initial stage of tooth damage. 

These results were confirmed by SEM-EDX observations. Indeed, SEM images of sound enamel showed smoother enamel surfaces in which the enamel prisms were intact. Conversely, these surfaces are more susceptible to the further progression of the demineralization process than the intact enamel surface, which contains a hypomineralized superficial layer [1,3]. WSL appeared as the initial loss of the structural integrity of the enamel, with some gaps between the crystallites and even deep grooves on an uneven surface, not uniformly demineralized, which varied in different parts of the lesions. This is due to the imbalance in favor of the demineralization process caused by the acids produced by bacteria, which destroy the crystalline structure. Indeed, the elemental composition identified by EDX clearly indicated a decrease in Ca and P percentage in the enamel surface with WSL, probably due to the loss of calcium and phosphate ions from the prismatic enamel structure.

The VMH test is one of the most commonly used and reliable methods to evaluate mineralization changes in hard dental tissues. Tooth surface microhardness reflects the physical–mechanical features of the tissue, strictly related to its microstructure, MD, and composition, as previously discussed. The results of the VMH tests clearly confirm the occurrence of a demineralization process in the WLSs, with a decrease in the mechanical resistance; in fact, the HV number passes from 318.2 ± 22.3 kg/mm^2^ in sound enamel to 125.6 ± 16.6 kg/mm^2^ in correspondence of the WLS surface. 

Some limitations to this in vitro study include the restricted sample size and the lack of direct clinical implications, but it is noteworthy to point out that the results reported here could be fundamental for paving the way for future basic and clinical research. In fact, this study defines a valid high-resolution method to deeply analyze hard dental tissues affected by initial carious lesions of the enamel. The comparison of 2D and 3D data obtained by different analytical techniques allows us to validate this multidisciplinary approach as a future method for correctly analyzing, homogenizing, and comparing results from different studies, shedding new light on the future development of possible minimally invasive treatments for these lesions. 

## 5. Conclusions

In this preliminary study, the coupling of µ-CT, RMS, SEM-EDX, and VMH has proven to be a valid analytical multidisciplinary approach to studying in vitro hard dental tissues, confirming the repeatability of this analysis. Based on the results, it can be concluded that WSLs are characterized by a reduction in enamel mineralization, as evidenced by the decrease in MD, enamel crystallinity, VMH values, mineral contents, and by the increase in the organic matrix. 

This method could have important implications in clinical practice since it allows the whole characterization of the area of interest, thus providing a deeper knowledge of these initial enamel lesions. Future studies with a larger number of samples also focused on the histological evaluation of WSL areas will be useful to confirm our results and to further observe modifications in dental hard tissues. 

## Figures and Tables

**Table 1:** Grey values (grey level) and corresponding mineral density (MD) calculated for WSL, enamel, and dentin. Data, obtained by the analysis of four different slices, are displayed as the mean ± standard deviation.

Tissue	Grey Level	MD (g/cm^3^)
WSL	118.75 ± 1.71	2.14 ± 0.021
Enamel	157.75 ± 0.50	2.52 ± 0.005
Dentin	95.00 ± 0.82	1.91 ± 0.008

## Data Availability

The datasets used and/or analyzed during the current study are available from the corresponding author on reasonable request.
